# Initial inpatient management of adolescents and young adults admitted with severe malnutrition due to anorexia nervosa: protocol for a systematic review

**DOI:** 10.1186/s40337-021-00389-6

**Published:** 2021-03-10

**Authors:** Stéphanie Proulx-Cabana, Danielle Taddeo, Olivier Jamoulle, Jean-Yves Frappier, Fannie Tremblay-Racine, Chantal Stheneur

**Affiliations:** 1 Pediatrics Department, Division of Adolescent Medicine, Sainte-Justine University Hospital Center, 3175 Côte-Sainte-Catherine Road, Montreal, Quebec H3T 1C5 Canada; 2 Librarian, Sainte-Justine University Hospital Center, 3175 Côte-Sainte-Catherine Road, Montreal, Quebec H3T 1C5 Canada; 3 CESP, Univ. Paris-Sud, UVSQ, INSERM U 1178, Université Paris-Saclay [Paris-Saclay University], 94805 Villejuif, France; 4 Clinique FSEF Varennes Jarcy, Fondation Sante des Etudiants de France, 91480 Varennes-Jarcy, France; 5 UFR des Sciences de la Santé Simone Veil [Simone Veil Health Science Training and Research Unit], Université de Versailles Saint-Quentin-en-Yvelines [Versailles Saint-Quentin-en-Yvelines University], Versailles, France

**Keywords:** Anorexia nervosa, Adolescent, Young adults, Inpatients, Systematic review

## Abstract

**Background:**

*Anorexia Nervosa* (AN) is a highly prevalent disease in adolescents and young adults (AYAs). The quality of initial inpatient medical management in a patient with severe malnutrition due to AN is crucial to prevent further medical instability. This review aims to inventory evidence regarding initial refeeding and management of AYAs with AN in an inpatient setting, in order to avoid medical complications.

**Methods:**

A systematic review will be conducted using PubMed, MEDLINE, All EBM Reviews, Embase, PsycINFO, Cochrane Database and CINAHL. The search strategy consists of terms related to anorexia, hospitalization and Pediatrics. Randomized controlled trials, case-control studies, cross-sectional and cohort studies will be included. Participants will include adolescents and adults 18–24 years old diagnosed with AN and meeting criteria for severe disease. The primary outcome will be any of the following complications: hypophosphatemia, refeeding syndrome, hypoglycemia, cardiac arrythmia, hepatic cytolysis or death. Data will be extracted and the risk of bias will be assessed by one author and reviewed by a second author. Results will be presented in a systematic narrative synthesis format. The quality of evidence for all outcomes will be evaluated using the GRADE methodology.

**Discussion:**

This systematic review will examine current evidence on initial inpatient refeeding and help to document effectiveness of initial inpatient management in AYAs with severe AN in avoiding further medical complications.

**Trial registration:**

This study is registered on PROSPERO under the reference number CRD42019123608.

**Supplementary Information:**

The online version contains supplementary material available at 10.1186/s40337-021-00389-6.

## Background

*Anorexia Nervosa* is the third most frequent chronic disease in adolescence, affecting up to 0.5% of adolescents [1, 2]. This disease is characterized by a multi-systemic impairment due to the metabolic complications of prolonged fasting and severe malnutrition. *Anorexia Nervosa* is also the psychiatric disease associated with the highest mortality rate, ranging from 2 to 8%, mainly attributable to cardiac complications and suicide [1, 2]. The risk of medical complications leading to death in a patient during initial inpatient stabilization period is proportional to the severity of the malnutrition [3]. The hospital admission of adolescents or young adults presenting with severe malnutrition secondary to *Anorexia Nervosa* constitutes an important challenge due to the fact that initial refeeding process can further increase their medical instability. The initial inpatient management of these patients is crucial in order to limit further weight lost and to monitor medical deterioration, including the development of a refeeding syndrome.

Multidisciplinary approaches, that include nutritional, psychological, physical and psychiatric interventions for patients with Anorexia Nervosa have been brought forward as an important component to promote recovery from AN and should be applied throughout the continuum of care from inpatient to outpatient modalities [4–6]. Moreover, a multidisciplinary approach has also been used during the initial medical stabilization of patients meeting the criteria for severe AN, when focus is put mainly on nutritional rehabilitation and clinical surveillance of complications [7]. However, as specialized multidisciplinary teams are few in number, management of patients medically unstable with severe AN is likely to be initiated within general medical or pediatric units [8]. The inexperience of these units in dealing with AN and especially severe cases can result in a lack of timely recognition of medical complications arising from the illness in itself or the initiation of refeeding [9]. This can lead to adverse events for these patients that could have been avoided if appropriate support from eating disorders experts had been provided in due time. By consolidating current evidence for the initial medial stabilization, this could help disseminate current evidence to those who lack a multidisciplinary team to support them, as well as identify areas where further research is necessary.

Even though there is a certain homogeneity in admission criteria in different guidelines, there is no clear consensus on optimal medical stabilization of newly admitted patients with A*norexia Nervosa.* In a 2003–2004 survey of North American physicians treating adolescent inpatients with *Anorexia Nervosa*, there were wide variations in caloric intake and preferred method of refeeding (meal based plan versus nasogastric tube feeding) [10]. Furthermore, only 37% of institutions used standardized protocol for initial refeeding [10]. In order to address this lack of clear evidence based clinical guidelines in the patients with severe instability, the *Royal College of Psychiatry* published in 2011 the Junior MARSIPAN (*Management of Really Sick Patients with Anorexia Nervosa),* a clinical guideline addressing children and adolescents with severe Anorexia Nervosa [11]). Although this guideline represents a great achievement in order to standardize inpatient admission practice, it is based on available practice guidelines at the time of publication and a literature review. So far, there has been no systematic review using recognized methodology, such as PRISMA guidelines, focused on initial admission protocols used in adolescents or young adults admitted with severe AN and how to prevent further medical instability.

The primary objective of the following systematic review is to inventory available evidence regarding initial medical management of adolescent and young adults (AYAs) presenting with severe malnutrition secondary to A*norexia Nervosa* and to assess the effects of this management on medical stability and complications. It is a secondary aim of the systematic review, with evidence permitting, to attempt to develop an evidence-based standardized admission protocol for adolescents with severe *Anorexia Nervosa* undergoing inpatient refeeding.

## Methods/design

### Eligibility criteria

Eligibility of papers to be included in this study will be evaluated according to the criteria outlined below and available in Table 1.

**Table 1:** Eligibility criteria for included studies

	Eligibility criteria
Study design	RCTs, prospective cohort studies, retrospective cohort studies, cross-sectional and case-control studies.
Age	Children and adolescents < 18 y.o. Young adult 18–24 y.o.
Gender	All gender
Diagnosis	Meeting criteria for Anorexia Nervosa based on DSM-5, DSMIV-R or ICD-10
Severity of disease	Severe malnutrition (BMI < 16 kg/m^2^ (adult) or percent median BMI ≤70% or Z-score ≤ − 3 SD (adolescents)) OR Moderate malnutrition (BMI 16–16.99 kg/m^2^ (adult) or percent median BMI 70–79% or Z-score − 2 to − 2.9 SD (adolescents)) AND Acute medical instability at admission
Admission	First and repeated admissions as long all episodes documented separately and other inclusion criteria are met
Interventions	Defining at least one the following element of an inpatient admission protocol intended for AYAs with AN: -Refeeding plan -Laboratory surveillance -Cardio-respiratory stability monitoring -Supplementation

#### Study design

Considering the paucity of randomized controlled trials (RCTs) in adolescents and young adults (AYAs) with A*norexia Nervosa,* in order to obtain an adequate sample, we will include randomized controlled trials (RCTs) as well as prospective and retrospective cohorts, cross-sectional and case-control studies. We will exclude case reports and case series from this review as we consider that quality of evidence expected from a very limited sample size will not allow us to conclude if the clinical evolution is a direct consequence of any admission inpatient protocol used.

#### Participants

We will include studies that had a study population of children and/or adolescents (< 18 years old) and/or young adults, defined as adults between the age of 19 and 24 years old. In studies addressing both adults and adolescents, we will include them only if data for adolescents and adults younger than 25 years old are reported separately from adults older than 25 years old. If separate data for adolescents and young adults, either reported separately or as a combined group distinct from older adults, are not available in a study that fulfills all the other inclusion criteria, we will try to contact the author to obtain data on this specific age group. Gender will not be an exclusion criterion and we will include studies considering all gendered patients. The diagnosis of AN will be based on established criteria from the Diagnostic and Statistical Manual of Mental Disorders fifth edition (DSM-5) or fourth-text revised edition (DSM-IVR) or from the International Classification of Diseases (ICD-10). We will exclude studies including multiple Eating Disorders diagnosis, such as *Bulimia Nervosa*, Avoidant/Restrictive Food Intake Disorder and Other Specified Feeding and Eating Disorders, in their study population unless they provide separate analysis of patients diagnosed with AN. The severity of AN is defined in the DSM-5 as patients who are considered severely malnourished as reflected by a BMI < 16 kg/m^2^ in adults or by a percent median BMI ≤70% based on the WHO growth charts or Z-score ≤ − 3 SD in adolescents [12]. However, we will consider also patients to have severe disease if they presented with moderate malnutrition (adult with a BMI of 16–16.99 kg/m^2^ or adolescent with a percent median BMI of 70–79% or Z-score of − 2 to − 2.9 SD [12]) concomitant with acute medical instability at admission, such as hypothermia, hypoglycemia, significant bradycardia, hypotension or electrolytes abnormalities. We will consider studies reporting first hospital admission as well as repeated hospital admissions as long as each episode of care is reported distinctly and that criteria for severity of AN is met in all different admissions.

#### Interventions

Studies describing at least one element of an inpatient admission protocol intended for AYAs with AN will be accepted. The refeeding plan should explicitly report the initial energy intake and refeeding methods preferred for the majority of patients, such as meal plan or nasogastric tube feeding, to be considered complete. Laboratory surveillance includes any mention of frequency of measurement of glycemia, phosphate, potassium, magnesium and calcium in patients or any other element considered relevant by based on extant literature and clinician experience. Bed-rest criteria, EKG frequency, admission to intensive care for surveillance or any other element considered relevant will be included in cardio-respiratory stability monitoring. Finally, we will evaluate if electrolytes and vitamin supplementation, including phosphate, potassium, thiamine and multivitamins, was mentioned as part of initial patient management. We will exclude studies that involved only outpatient or partial hospitalization management.

### Search strategy

Literature search strategies will be developed using medical subject headings (MeSH) and text words related to *Anorexia.* Systematic searches of the databases PubMed (NLM), Ovid Medline, Ovid All EBM Reviews, Ovid Embase, Ovid PsycINFO, Cochrane Database and EBSCO CINAHL will be completed by a librarian of our Hospital with special training and skills in literature searches. A draft PubMed search strategy is included in Additional file 1. Once the PubMed strategy is finalized, it will be adapted to the syntax and subject headings of the other databases. If further information is needed for some identified studies, we will contact the authors in order to try to obtain adequate information. In order to ensure that all relevant material has been identified, we will also scan the reference lists of included studies or relevant reviews identified through the search.

The search will include both qualitative and quantitative studies. No study design or date limit will be imposed. However, we will limit the search to articles reported either in French or English language.

The search will be updated toward the end of the review in order to retrieve any additional relevant articles that might have been published after the initial search date.

### Outcome measures

#### Primary outcomes

The primary outcome of this systematic review is the reported frequency of adverse events during the initial stabilization period following admission to an inpatient unit. We will consider that the initial stabilization period extends up to 7 days after admission. We will further focus on adverse events that are related to the elements included in the description of the admission protocol given by each study. We plan to analyze separately all these outcomes whether the patient was initially refed at higher caloric intake (> 1400 kcal/day or > 40 kcal/kg/day) or lower caloric intake (< 1400 kcal/day or < 40 kcal/kg/day) (11). The adverse events of interest are:

-Hypophosphatemia: characterized as low phosphate levels in the blood. We will consider: a) the appearance of a new hypophosphatemia or the deterioration of a hypophosphatemia that was present at admission with the initiation of refeeding as significant, b) the prevalence of hypophosphatemia if systematic supplementation with phosphorus is initiated at admission.

-Refeeding syndrome: Even though no clear definition is used in the literature to define the refeeding syndrome [13], we will consider the presence of refeeding syndrome if there is concomitant presence of hypophosphatemia and/or any other electrolytic imbalances with clinical symptoms compatible with refeeding including: a) hypoglycemia, b) cardiac failure, c) oedema, d) hepatic failure, e) delirium, f) arrhythmia, g) rhabdomyolysis, h) hepatic failure.

-Hypoglycemia: characterized as low blood sugar levels in the blood. We will consider separately: a) preprandial hypoglycemia, b) postprandial hypoglycemia, c) hypoglycemia refractory to suggested applied correction protocol.

-Cardiac arrhythmia: characterized as non-sinusal rhythm on EKG. We will consider any new arrhythmia that developed after admission.

-Hepatic cytolysis: we will consider any elevated transaminase levels defined as ALT and/or AST > 40 U/L: a) developed during the refeeding process, b) initially elevated that deteriorated during the immediate refeeding process.

-Death: we will only consider death that took place during the stabilization period.

Another primary outcome of this systematic review is to establish the effectiveness of the examined protocols for the initial stabilization period as previously defined of 7 days: We will evaluate this based on a) the weight trajectory in the first 7 days b) frequency of adverse effects reported in lights of each elements of the protocol c) If possible, comparison of the frequency of adverse effects for protocols with similar components d) time for patient to reach medical stabilization if hospitalization extends over initial medical stabilization period d) time for patient to be discharged if hospitalization ends once medical stabilization is reached.

#### Secondary outcomes

For secondary outcomes, we will evaluate different parameters that can be related to initial management but don’t constitute direct complications of aforementioned adverse events:

- Bradycardia: characterized as a heart rate slower than 60 beats per minutes. We will evaluate the time to achieve a heart rate over 50 beats per minute as it is the most recommended cut-off rate to which admission is warranted.
- Prolonged QT interval: we will evaluate: a) proportion of prolonged QT interval at admission, b) medication associated with prolonged QT interval, c) time to complete recovery of the prolonged QT interval.
- Gastro-intestinal complications: during initial refeeding, we will consider: a) all acute gastric dilatation reported, b) all superior mesenteric artery syndrome reported.
- Initial weight lost: we will evaluate the initial weight lost with initial refeeding caloric intake suggested in the study protocol.
- Effectiveness of the laboratory surveillance suggested for refeeding syndrome: when obtainable, we will evaluate this outcome by looking at the reported occurrence of electrolyte imbalance in terms of: a) timing of occurrence since beginning of refeeding, b) frequency of occurrence considering initial caloric intake, method of refeeding (meal-plan, continuous or intermittent nasogastric feeding) and use of systematic supplementation.
- Side effects of prophylactic supplementation when used: we will evaluate any reported side effect associated with supplementation of: a) Phosphate, b) Potassium, c) Magnesium, d) Calcium, e) Glucose, f) Thiamine, g) Multivitamins.

### Study selection

Literature search results will be imported into a commercial reference management software package (EndNote version X8.2, 2018, Clarivate Analytics) and all duplicates will be excluded.

One author will review the title and abstract of all the references to identify potentially eligible studies. We will obtain full text articles for all titles that appear to meet inclusion criteria or where there is any uncertainty. The full articles will be examined by two authors independently to decide whether they meet the inclusion criteria. We will seek additional information from study authors where useful to resolve eligibility. We will resolve disagreements through discussion. If agreement is not achieved between the two authors through discussion, another co-author will act as a third-party to reached a final decision on eligibility. We will record the reasons for which a study will be excluded from the systematic review.

### Data extraction

For data extraction, we will use a standardized form. This form will be developed by the systematic review team by adapting the Cochrane data collection form [14] for intervention review to the specific requirements of this study. To ensure consistency across reviewers, we will conduct a calibration exercise before starting the review. Data collection will be performed by one author of the systematic review.

Data extracted will consists of:

- *Publications details:* authors, title and publication date.
- *Study design*: aim of study, study design, if an RCT description of the blinding method if present, start and end date of the study, diagnostic criteria upon which the diagnosis of *Anorexia Nervosa* was based, methods of recruitment, length of follow-up.
- *Patients characteristics:* age, gender, number of participants, number of admissions, severity of the disease at admission, baseline BMI, baseline median BMI, baseline weight, initial medical instability, co-morbidities.
- *Intervention:*
  - Refeeding plan: including initial daily energy intake at initiation of refeeding, progression in intake and maximum number of daily calories consumed, macronutrient distribution, refeeding methods including type of refeeding solution if a nasogastric tube is used, duration of the intervention, total daily fluid intake.
  - Laboratory surveillance: Including frequency of measurement of basic blood tests (CBC, liver function tests, urea, creatinine, TSH, glycemia, phosphate, sodium, potassium, magnesium, calcium).
  - Protocol for hypoglycemia.
  - Cardio-respiratory stability monitoring: including bed-rest criteria based on bradycardia, EKG frequency, criteria for admission to the intensive care unit for surveillance, standardized use of telemetry monitor, arterial blood pressure measure frequency and use of orthostatic arterial blood pressure.
  - Electrolytes and vitamin supplementation: including serum level cut-off for supplementation of phosphate, potassium, calcium and magnesium, dosage use for these supplementations, prophylactic use of phosphate, thiamine or multivitamins.
- *Study results:* findings related to the primary and secondary outcomes as described in the previous section.

A second author will review all the data collection forms completed and resolve disagreements by discussion with the first author. Once more, if agreement is not achieved between the two authors through discussion, another co-author will act as a third-party to achieve a final decision. We will contact study authors by email in case of uncertainties. If information is still not obtained after 3 attempts to reach the study authors, we will consider this information missing.

### Risk of bias

Since we are planning to include RCTs and non-randomized studies, such as cohort studies and case-control studies, we will be using two different tools to evaluate the risk of bias in our systematic review.

For RCTs, we will assess the possible risk of bias for each study using the Cochrane Collaboration tool for assessing the risk of bias [15]. We will collect information for all items included in this tool: sequence generation, allocation concealment, blinding, incomplete outcome data and selective outcome reporting. From the extracted information, we will rate each of these categories as “high risk” or “low risk”. If there is insufficient information in one category to evaluate the risk, we will classify the risk as “unclear risk”. The judgement on risk will be performed by one author using this tool. A second author will review all completed risk assessments. We will resolve disagreements between the two authors by discussion and use a third author, if necessary, to resolve disagreements.

For non-randomized studies, we will assess risk of bias using the Newcastle-Ottawa scale (NOS) for case-control and cohort studies [16]. Each study will be evaluated by one author and attributed a score over 9 points on items included in the form. We will consider studies with 7 points or higher as high quality and studies with 5–6 points as moderate quality. Once again, a second author will review all completed scales and, in case of disagreements, we will resolve it through discussion between the two authors. A third author will again be involved if disagreement persists between the two authors despite discussion.

### Data synthesis

Since available literature on *Anorexia Nervosa* in general is principally composed of non-randomized studies, we expect to include only a few, if any, RCTs in this systematic review. We do not expect to have enough RCTs to conduct a meta-analysis. We will use a systematic narrative synthesis instead. In the synthesis, we will provide information in the text and tables to summarize and explain the characteristics and findings of the included studies. We will present all results on our first objective, which was to assess the effects of initial medical management of adolescents and young adults admitted with severe *Anorexia Nervosa* on medical stability and complications. We will synthesize data obtained into main and additional outcomes. We hope to be able to present separate data for these two age groups, but if only combined information is obtained, we might need to present data without this distinction between the two groups. As we expect a small number of studies to fulfill the inclusion criteria and we expect studies on *Anorexia Nervosa* to have at least moderate risk of bias, we will not systematically exclude results from high risk of bias study, but we will comment on the risk of bias for each included study and consider it in the conclusion that will arise from our systematic review.

If the evidence gathered in this systematic review allow to draw conclusion on expected best practices, we will aim to develop an evidence-based standardized admission protocol for adolescents with severe *Anorexia Nervosa*. We will integrate a complete version of this protocol as an Appendix of this systematic review if this second objective can be fully completed.

### Meta-bias (es)

As we expected to have only a few RCTs, the evaluation of meta-bias, such as publication bias and outcome reporting bias, will be difficult to evaluate in the absence of a published protocol register in case-control studies and cohort studies. In that case, no evaluation of meta-biases will be performed. However, for RCTs included, we will evaluate, using the Clinical Trial Register Platform of the World Health Organization, if there are any differences in outcomes reported between the published study and study protocol. We will also evaluate reporting bias by determining if recruitment started before the publication of the study protocol.

### Confidence in cumulative evidence

The quality of evidence for all outcomes will be evaluated using the Grading of Recommendations Assessment, Development and Evaluation working group methodology (GRADE). The quality will be reported as high (further research is very unlikely to change our confidence in the estimate of effect), moderate (further research is likely to have an important impact on our confidence in the estimate of effect and may change the estimate), low (further research is very likely to have an important impact on our confidence in the estimate of effect and is likely to change the estimate) or very low (very uncertain about the estimate of effect).

## Discussion

One of the strengths of this systematic review is its use of a transparent and systematic methodology in order to have a better understanding of most recent evidence in terms of medical management, which had never been done to our knowledge. Also, if we succeed in establishing a clinical protocol based on actual evidence, it can further help in standardizing best practices for medical teams treating AYAs with severe AN.

The challenges we expect to encounter is the paucity of studies with an RCT design in this specific population, considering that the literature in AN in general has only a few RCTs. We will probably need to rely more on observational design of studies, which is associated with a larger risk of introducing bias. This might limit our ability to fulfill the second aim of this study as evidence found might not be robust enough to allow for a clear admission protocol to be created.

Another limitation that we expect is that, since we only considered studies on inpatients and there are several modalities of treatment used for patients with AN (ex: residential treatment, partial-hospitalization, etc.), our findings might not be generalizable to other treatment options for severe AN. However, since the risk of initial medical instability is greater in this population, we expect that most of these patients will meet criteria for admission to inpatient facilities. Finally, we hope to be able to obtain separate data for adolescents and young adults, but there is a possibility that we only obtain combined data for these age groups, limiting our ability to analyze for specific age group. The reason for trying to get distinct data is that the care of young adults with AN is subjected to very different legal frameworks than adolescents younger than 18 years old [6]. Also, we believe that care for those younger than 18 years old is most likely to be offered by pediatrician which reflects a different practice setting to those who are of adult age. However, as advocacy for more inclusive care and focus that extends further than 18 years old has been highlighted recently [17], we believe that reporting this as a combined group would still bring essential highlight on current evidence.

Nonetheless, this systematic review will be a great opportunity to attempt to summarize actual evidence on the initial management of AYAs admitted for severe AN and to reflect on areas for which further good quality research is needed to improve patient care.

## Supplementary Information

**Additional file 1.**

**Additional file 2.**

## Data Availability

Not applicable.

## Abbreviations

ALT: Alanine aminotransferase
AN: Anorexia Nervosa
AST: Aspartate amino transferase
AYA: Adolescent and Young Adult
BMI: Body-mass index
CBC: Complete blood count
DSM: Diagnostic and Statistical Manual of Mental Disorders
EKG: Electrocardiogram
GRADE: Grading of Recommendations Assessment, Development and Evaluation
ICD: International Classification of Diseases
MARSIPAN: Management of Really Sick Patients with Anorexia Nervosa
MeSH: Medical subject headings
NOS: Newcastle-Ottawa Scale
PRISMA: Preferred Reporting Items for Systematic Reviews and Meta-Analyses
PROSPERO: Prospective Register of Systematic Reviews
RCT: Randomized controlled trials
SD: Standard deviation
TSH: Thyroid stimulating hormone
WHO: World Health Organization
