# Supplementary material for: Initial inpatient management of adolescents and young adults admitted with severe malnutrition due to anorexia nervosa: protocol for a systematic review
Source: J Eat Disord. 2021 Mar 10;9:36. doi: 10.1186/s40337-021-00389-6 (PMC7944889; doi:10.1186/s40337-021-00389-6)
Supplement: Supplementary file 1 — Additional file 1. [file 40337_2021_389_MOESM1_ESM.pdf]

Additional file 1: Pubmed Search strategy applied

1	Anorexia	Anorexia[MH] OR Anorexia Nervosa[MH] OR anorexi*[TIAB] OR anorexi*[OT]
2	Hospitalisation	Hospitalization[MH] OR Patient Admission[MH] OR Inpatients[MH] OR "Adolescent, Hospitalized"[MH] OR "Child, Hospitalized"[MH] OR hospitalization*[TIAB] OR hospitalisation*[TIAB] OR hospital stay*[TIAB] OR hospitalized[TIAB] OR hospitalised[TIAB] OR admission*[TIAB] OR inpatient*[TIAB] OR "in-hospital patient"[TIAB] OR "in-hospital patient'"[TIAB] OR "in-hospital patients"[TIAB] OR "in-hospital patient's"[TIAB] OR "in-patient"[TIAB] OR "in-patient'"[TIAB] OR "in-patients"[TIAB] OR "in-patient's"[TIAB] OR "hospital patient"[TIAB] OR "hospital patient'"[TIAB] OR "hospital patients"[TIAB] OR "hospital patient's"[TIAB] OR "admitted to the hospital"[TIAB] OR hospitalization*[OT] OR hospitalisation*[OT] OR hospital stay*[OT] OR hospitalized[OT] OR hospitalised[OT] OR admission*[OT] OR inpatient*[OT] OR "in-hospital patient"[OT] OR "in-hospital patient'"[OT] OR "in-hospital patients"[OT] OR "in-hospital patient's"[OT] OR "in-patient"[OT] OR "in-patient'"[OT] OR "in-patients"[OT] OR "in-patient's"[OT] OR "hospital patient"[OT] OR "hospital patient'"[OT] OR "hospital patients"[OT] OR "hospital patient's"[OT] OR "admitted to the hospital"[OT]
3	Pediatrics	Child[MH] OR Adolescent[MH] OR Intensive Care Units, Pediatric[MH:NOEXP] OR Hospitals, Pediatric[MH] OR Nurses, Pediatric[MH:NOEXP] OR Pediatrics[MH:NOEXP] OR Pediatric Emergency Medicine[MH] OR Pediatricians[MH:NOEXP] OR "Child, Hospitalized"[MH] OR "Adolescent, Hospitalized"[MH] OR Young Adult[MH] OR child[TIAB] OR child'[TIAB] OR childs[TIAB] OR child's[TIAB] OR children*[TIAB] OR childhood*[TIAB] OR kid[TIAB] OR kid'[TIAB] OR kids[TIAB] OR kid's[TIAB] OR boy[TIAB] OR boy'[TIAB] OR boys[TIAB] OR boy's[TIAB] OR girl[TIAB] OR girl'[TIAB] OR girls[TIAB] OR girl's[TIAB] OR schoolchild*[TIAB] OR juvenil*[TIAB] OR preadolescen*[TIAB] OR youth*[TIAB] OR adolescen*[TIAB] OR teen[TIAB] OR teen'[TIAB] OR teens[TIAB] OR teen's[TIAB] OR teenage*[TIAB] OR puber[TIAB] OR puber'[TIAB] OR pubers[TIAB] OR puber's[TIAB] OR pubert*[TIAB] OR pubescen*[TIAB] OR high school*[TIAB] OR highschool*[TIAB] OR secondary school*[TIAB] OR paediatric*[TIAB] OR pediatric*[TIAB] OR PICU*[TIAB] OR "young adult"[TIAB] OR "young adult'"[TIAB] OR "young adults"[TIAB] OR "young adult's"[TIAB] OR "prime adult"[TIAB] OR "prime adult'"[TIAB] OR "prime adults"[TIAB] OR "prime adult's"[TIAB] OR child[OT] OR child'[OT] OR childs[OT] OR child's[OT] OR children*[OT] OR childhood*[OT] OR kid[OT] OR kid'[OT] OR kids[OT] OR kid's[OT] OR boy[OT] OR boy'[OT] OR boys[OT] OR boy's[OT] OR girl[OT] OR girl'[OT] OR girls[OT] OR girl's[OT] OR schoolchild*[OT] OR juvenil*[OT] OR preadolescen*[OT] OR youth*[OT] OR adolescen*[OT] OR teen[OT] OR teen'[OT] OR teens[OT] OR teen's[OT] OR teenage*[OT] OR puber[OT] OR puber'[OT] OR pubers[OT] OR puber's[OT] OR pubert*[OT] OR pubescen*[OT] OR high school*[OT] OR highschool*[OT] OR secondary school*[OT] OR paediatric*[OT] OR pediatric*[OT] OR PICU*[OT] OR "young adult"[OT] OR "young adult'"[OT] OR "young adults"[OT] OR "young adult's"[OT] OR "prime adult"[OT] OR "prime adult'"[OT] OR "prime adults"[OT] OR "prime adult's"[OT]
4	Combination et limitations	(#1 AND #2 AND #3) AND (english[LA] OR french[LA])
